# Emergence of Lie Symmetries in Functional Architectures Learned by CNNs

**DOI:** 10.3389/fncom.2021.694505

**Published:** 2021-11-22

**Authors:** Federico Bertoni, Noemi Montobbio, Alessandro Sarti, Giovanna Citti

**Affiliations:** ^1^Sorbonne Université, Paris, France; ^2^Dipartimento di Matematica, Università di Bologna, Bologna, Italy; ^3^Centre d'analyses et de Mathematiques Sociales, CNRS, EHESS, Paris, France; ^4^Neural Computation Laboratory, Center for Human Technologies, Istituto Italiano di Tecnologia, Genova, Italy

**Keywords:** lie symmetries, CNN-convolutional neural network, primary visual cortex (V1), lateral connection, lateral geniculate, sub-Riemannian geometries

## Abstract

In this paper we study the spontaneous development of symmetries in the early layers of a Convolutional Neural Network (CNN) during learning on natural images. Our architecture is built in such a way to mimic some properties of the early stages of biological visual systems. In particular, it contains a pre-filtering step ℓ^0^ defined in analogy with the Lateral Geniculate Nucleus (LGN). Moreover, the first convolutional layer is equipped with lateral connections defined as a propagation driven by a learned connectivity kernel, in analogy with the horizontal connectivity of the primary visual cortex (V1). We first show that the ℓ^0^ filter evolves during the training to reach a radially symmetric pattern well approximated by a Laplacian of Gaussian (LoG), which is a well-known model of the receptive profiles of LGN cells. In line with previous works on CNNs, the learned convolutional filters in the first layer can be approximated by Gabor functions, in agreement with well-established models for the receptive profiles of V1 simple cells. Here, we focus on the geometric properties of the learned lateral connectivity kernel of this layer, showing the emergence of orientation selectivity w.r.t. the tuning of the learned filters. We also examine the short-range connectivity and association fields induced by this connectivity kernel, and show qualitative and quantitative comparisons with known group-based models of V1 horizontal connections. These geometric properties arise spontaneously during the training of the CNN architecture, analogously to the emergence of symmetries in visual systems thanks to brain plasticity driven by external stimuli.

## 1. Introduction

The geometry of the visual system has been widely studied over years, starting from the first celebrated descriptions given by Hubel and Wiesel (1962) and Hubel (1987) and advancing with a number of more recent geometrical models of the early stages of the visual pathway, describing the functional architectures in terms of group invariances (Hoffman, 1989; Citti and Sarti, 2006; Petitot, 2008). Some works have also focused on reproducing processing mechanisms taking place in the visual system using these models—e.g., detection of perceptual units in Sarti and Citti (2015), image completion in Sanguinetti et al. (2008).

On the other hand, relations between Convolutional Neural Networks (CNNs) and the human visual system have been proposed and studied, in order to make CNNs even more efficient in specific tasks (see e.g., Serre et al., 2007). For instance, in Yamins et al. (2015) and Yamins and DiCarlo (2016) the authors have been able to study several cortical layers by focusing on the encoding and decoding ability of the visual system, whereas in Girosi et al. (1995), Anselmi et al. (2016), and Poggio and Anselmi (2016) the authors have studied some invariance properties of CNNs. A parallel between horizontal connectivity in the visual cortex and lateral connections in neural networks has also been proposed in some works (see e.g., Liang and Hu, 2015; Spoerer et al., 2017; Sherstinsky, 2020). Recently, other biologically-inspired modifications of the classical CNN architectures have been introduced (Montobbio et al., 2019; Bertoni et al., 2022).

In this paper, we combine the viewpoints of these strands of research by studying the emergence of biologically relevant geometrical symmetries in the early layers of a CNN architecture. We focus on drawing a parallel between the patterns learned from natural images by specific computational blocks of the network, and the symmetries arising in the functional architecture of the Lateral Geniculate Nucleus (LGN) and the primary visual cortex (V1). After recalling in section 2 the main symmetries of the visual cortex, in section 3 we introduce an architecture similar to standard CNN models found in the literature, except for two main modifications. First, we insert a pre-filtering layer ℓ^0^ composed of one single filter shifting over the whole input image—corresponding to a layer of neurons whose receptive profiles are assumed to have all the same shape. Second, we introduce convolutional lateral connections acting on the feature space of the first network layer ℓ^1^. Such connections are defined in analogy with a Wilson-Cowan-type evolution equation with a plastic connectivity kernel weighting the strength of pairwise interactions.

As it is detailed in section 4, the filter learned by layer ℓ^0^ has a radially symmetric shape similar to LGN receptive profiles, extending the results of Bertoni et al. (2022) to a more complex architecture. section 5 focuses on ℓ^1^ receptive profiles, showing Gabor-like shapes as expected from previous work (Krizhevsky et al., 2009; Zeiler and Fergus, 2014). However, thanks to the pre-filtering layer ℓ^0^, our layer ℓ^1^ only contains filters sharply tuned for orientation, with no radially symmetric filters. In addition, by fitting the filters in the first ℓ^1^ layer with Gabor functions, we are able to use their parameters of position and orientation as coordinates for the ℓ^1^ layer itself. This provides a basis to study the geometry of ℓ^1^ lateral connections in what follows and to compare it with existing geometric models of the cortical long range connectivity in the Lie group of rotation and translation (Citti and Sarti, 2006). Indeed, in section 6 we describe the relationship between the learned distribution of position and orientation tuning of layer ℓ^1^ neurons and, in these new coordinates, the strength of lateral connectivity between two neurons through a learned kernel *K*^1^. As a consequence, the learned kernels and filters are re-mapped into the ℝ^2^ × *S*^1^ feature space. The last part of the section is devoted to studying the short-range connectivity as a function of orientation, and association fields induced by the resulting anisotropic connectivity kernel, comparing them with the curves of edge co-occurrence of Sanguinetti et al. (2010). In this way we prove the spontaneous emergence of Lie symmetries in the proposed biologically inspired CNN, as the symmetries encoded in the learned weights.

## 2. Group Symmetries in the Early Visual Pathway

Over the years, the functional architecture of the early visual pathway has often been modeled in terms of geometric invariances arising in its organization, e.g., in the spatial arrangement of cell tuning across retinal locations, or in the local configuration of single neuron selectivity. Certain classes of visual cells are shown to act, to a first approximation, as linear filters on an optic signal: the response of one such cell to a visual stimulus *I*, defined as a function on the retina, is given by the integral of *I* against a function ψ, called the *receptive profile* (RP) of the neuron:


(1) $$\begin{array}{l} z:=\int I\left(x,y\right)\psi \left(x,y\right)dxdy. \end{array}$$

This is the case for cells in the Lateral Geniculate Nucleus (LGN) and for simple cells in the primary visual cortex (V1) (see e.g., Citti and Sarti, 2006; Petitot, 2008). Both types of cells are characterized by locally supported RPs, i.e., they only react to stimuli situated in a specific retinal region. Each localized area of the retina is known to be associated with a bank of similarly tuned cells (see e.g., Hubel and Wiesel, 1977; Sarti and Citti, 2015), yielding an approximate invariance of their RPs under translations.

The set of RPs of cells is typically represented by a bank of linear filters ${\left\{\psi_{p}\right\}}_{p\in G}\subseteq L^{2}\left({\mathbb{R}}^{2}\right)$ (for some references see e.g., Citti and Sarti, 2006; Petitot, 2008; Sarti et al., 2008). The feature space G is typically specified as a group of transformations of the plane $\left\{T_{p},p\in G\right\}$ under which the whole filter bank is invariant: each profile ψ_*p*_ can be obtained from any other profile ψ_*q*_ through the transformation *T*_*p* − *q*_. The group G often has the product form ${\mathbb{R}}^{2}\times F$, where the parameters $\left(x_{0},y_{0}\right)\in {\mathbb{R}}^{2}$ determine the retinal location where each RP is centered, while f∈F encodes the selectivity of the neurons to other local features of the image, such as orientation and scale.

### 2.1. Rotational Symmetry in the LGN

A crucial elaboration step for human contrast perception is represented by the processing of retinal inputs via the radially symmetric families of cells present in the LGN (Hubel and Wiesel, 1977; Hubel, 1987). The RPs of such cells can be approximated by a Laplacian of Gaussian (LoG):


(2) $$\begin{array}{l} \psi_{LoG}\left(x,y\right)=-\frac{1}{\pi \sigma^{4}}\left[1-\frac{x^{2}+y^{2}}{2\sigma^{2}}\right]e^{-\frac{x^{2}+y^{2}}{2\sigma^{2}}}, \end{array}$$

where σ denotes the standard deviation of the Gaussian function (see e.g., Petitot, 2008).

### 2.2. Roto-Translation Symmetries in V1 Feedforward and Lateral Connectivity

The invariances in the functional architecture of V1 have been described through a variety of mathematical models. The sharp orientation tuning of simple cells is the starting point of most descriptions. This selectivity is not only found in the response of each neuron to feedforward inputs, but is also reflected in the *horizontal* connections taking place between neurons of V1. These connections show facilitatory influences for cells that are similarly oriented; moreover, the connections departing from each neuron spread anisotropically, concentrating along the axis of its preferred orientation (see e.g., Bosking et al., 1997).

An established model for V1 simple cells is represented by a bank of Gabor filters ${\left\{\psi_{x_{0},y_{0},\theta ,\sigma }\right\}}_{\left(x_{0},y_{0},\theta ,\sigma \right)\in {\mathbb{R}}^{2}\times S^{1}\times {\mathbb{R}}_{+}^{2}}$ built by translations *T*_(_*x*__0_, *y*_0_)_, rotations *R*_θ_ and dilations *D*_σ_*x*_, σ_*y*__ of the filter


(3) $$\begin{array}{l} \psi_{0,0,0,1}\left(x,y\right)=Ae^{-\frac{x^{2}+y^{2}}{2}}\cos\left(2\pi fx+\phi \right), \end{array}$$

where *A* is the amplitude and *f* is the frequency of the filter and ϕ is the phase which indicates if the Gabor filter is even or odd. See e.g., Daugman (1985) and Lee (1996).

The evolution in time *t* ↦ *h*(*p, t*) of the activity of the neural population at $p\in {\mathbb{R}}^{2}\times F$ is assumed in Bressloff and Cowan (2003) to satisfy a Wilson-Cowan equation (Wilson and Cowan, 1972):


$$\partial_{t}h\left(p,t\right)=-\alpha \;h\left(p,t\right)+s\left(\int K\left(p,p^{\prime }\right)h\left(p^{\prime },t\right)dp^{\prime }+z\left(p,t\right)\right).$$

Note that, if *K*(*p, p*′) is of the special form *K*(*p* − *p*′), then the integral in Equation (4) becomes a convolution as follows


(4) $$\begin{array}{l} \partial_{t}h\left(p,t\right)=-\alpha \;h\left(p,t\right)+s\left(K*h+z\right). \end{array}$$

Here, *s* is an activation function; α is a decay rate; *z* is the feedforward input corresponding to the response of the simple cells in presence of a visual stimulus, as in Equation (1); and the kernel *K* weights the strength of horizontal connections between *p* and *p*′. The form of this connectivity kernel has been investigated in a number of studies employing differential geometry tools. A breakthrough idea in this direction has been that of viewing the feature space as a fiber bundle with basis ℝ^2^ and fiber F. This approach first appeared in the works of Koenderink and van Doom (1987) and Hoffman (1989). It was then further developed by Petitot and Tondut (1999) and Citti and Sarti (2006). In the latter work, the model is specified as a sub-Riemannian structure on the Lie group ℝ^2^ × *S*^1^ by requiring the invariance under roto-translations. Other works extended this approach by inserting further variables such as scale, curvature, velocity (see e.g., Sarti et al., 2008; Barbieri et al., 2014; Abbasi-Sureshjani et al., 2018).

As described in Bosking et al. (1997) the cells connected with the same retinal region and with different preferred orientations form *hypercolumnar* modules, organized in “pinwheel” arrangements. At each retinal location the most excited cell is selected, leading to the so-called *non-maximal suppression* principle. This behavior is the result of a short-range connectivity that induces excitation between cells with close orientation and inhibition between cells with different orientation. This kind of connectivity has been modeled as a “Mexican hat”-like function in Bressloff and Cowan (2003).

**Figure 1:**
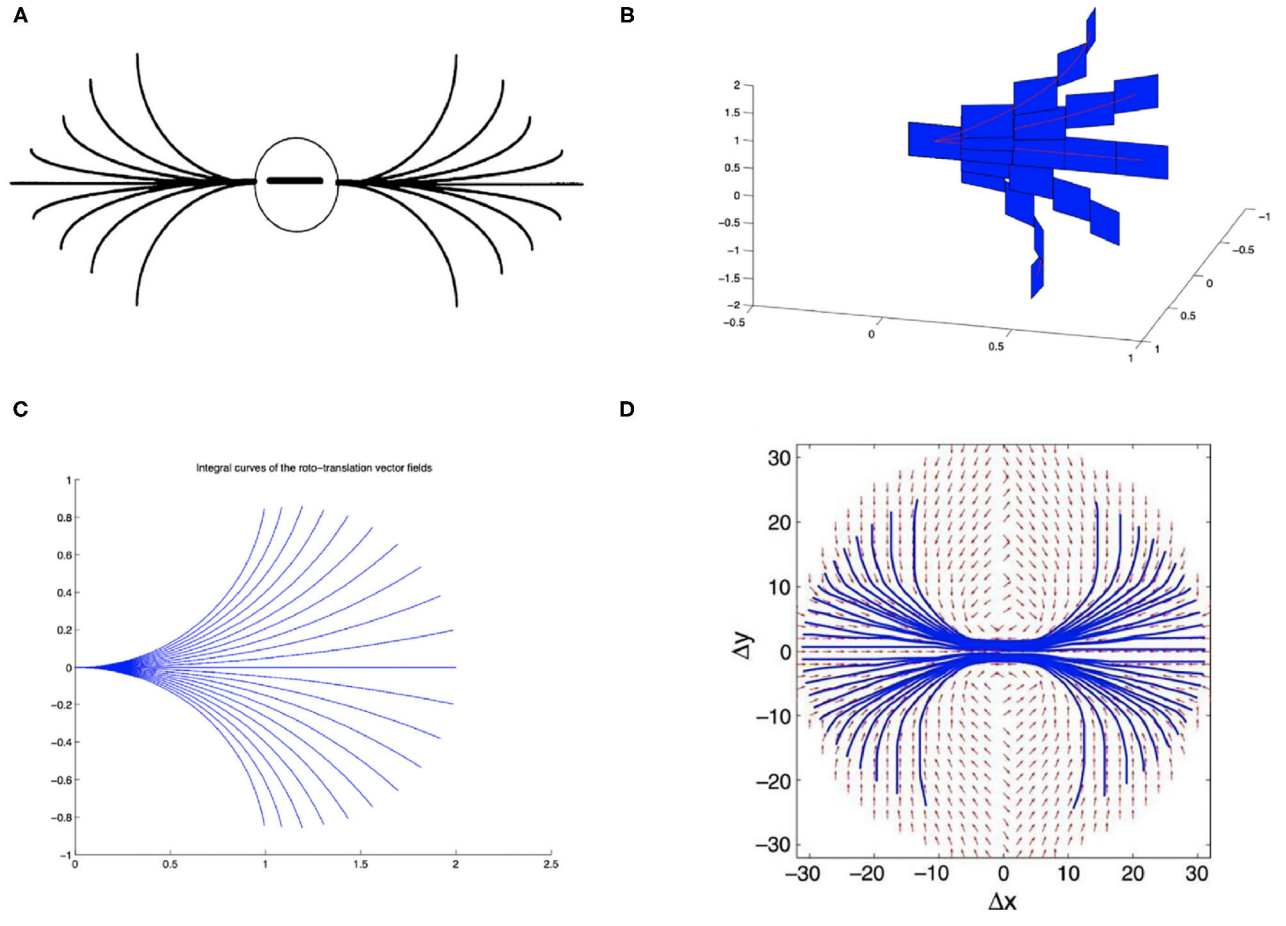
**(A)** Association fields from the experiment of Field, Hayes and Hess (Field et al., 1993). **(B)** 3D representation of the association field with contact planes of integral curves of the fields (Equation 5) with varying values of the parameter *k*, from Citti and Sarti (2006). **(C)** Integral curves of the fields (Equation 5) with varying *k*, from Citti and Sarti (2006). **(D)** The vector field of unitary vectors oriented with the maximal edge co-occurrence probability (red) with superimposed its integral curves (blue), from Sanguinetti et al. (2010).

On the other hand, the long-range horizontal connections of V1 allow cells belonging to different hypercolumns, but with similar orientation, to interact with each other. As a result, propagation of the visual signal along long-range horizontal can justify contour completion, i.e., the ability to group local edge items into extended curves. This perceptual phenomenon has been described through *association fields* (Field et al., 1993), characterizing the geometry of the mutual influences between oriented local elements. See Figure 1A from the experiment of Field, Heyes and Hess. Association fields have been characterized in Citti and Sarti (2006) as families of integral curves of the two vector fields


(5) $$\begin{array}{l} \vec{X_{1}}=\left(\cos\theta ,\sin\theta ,0\right),\;\vec{X_{2}}=\left(0,0,1\right) \end{array}$$

generating the sub-Riemannian structure on ℝ^2^ × *S*^1^. Figure 1B shows the 3-D constant coefficients integral curves of the vector fields (Equation 5) and Figure 1C their 2-D projection. These integral curves are the solution of the following ordinary differential equation:


$$\begin{array}{l} \gamma^{\prime }\left(t\right)=X_{1}\left(\gamma \left(t\right)\right)+kX_{2}\left(\gamma \left(t\right)\right). \end{array}$$

The curves starting from (0, 0, 0) can be rewritten explicitly in the following way:


(6) $$\begin{array}{l} \begin{array}{c} x=\frac{1}{k}sin\left(kt\right),\;y=\frac{1}{k}\left(1-cos\left(kt\right)\right),\;\theta =kt. \end{array} \end{array}$$

while integral curves starting from a general point (*x*_0_, *y*_0_, θ_0_) can be generated from equations (6) by translations *T*_(_*x*__0_, *y*_0_)_ and rotations *R*_θ_.

The probability of reaching a point (*x, y*, θ) starting from the origin and moving along the stochastic counterpart of these curves can be described as the fundamental solution of a second order differential operator expressed in terms of the vector fields $\vec{X_{1}}$, $\vec{X_{2}}$. This is why the fundamental solution of the sub-Riemannian heat kernel or Fokker Planck (FP) kernel have been proposed as alternative models of the cortical connectivity. This perspective based on connectivity kernels was further exploited in Montobbio et al. (2020): the model of the cortex was rephrased in terms of metric spaces, and the long range connectivity kernel directly expressed in terms of the cells RPs: in this way a strong link was established between the geometry of long range and feedforward cortical connectivity. Finally, in Sanguinetti et al. (2010) a strong relation between these models of cortical connectivity and statistics of edge co-occurrence in natural images was proved: the FP fundamental solution is indeed a good model also for the natural image statistics. In addition, starting from a connectivity kernel parameterized in terms of position and orientation, in Sanguinetti et al. (2010) they obtained the 2-D vector field represented in red in Figure 1D, whose integral curves (depicted in blue) provide an alternative model of association fields, learned from images.

## 3. The Underlying Structure: A Convolutional Neural Network Architecture

In this section we introduce the network model that will constitute the fundamental structure at the basis of all subsequent analyses. The main architecture consists of a typical Convolutional Neural Network (CNN) for image classification (see e.g., Lawrence et al., 1997; LeCun et al., 1998). CNNs were originally designed in analogy with information processing in biological visual systems. In addition to the hierarchical organization typical of deep architectures, translation invariance is enforced in CNNs by local convolutional windows shifting over the spatial domain. This structure was inspired by the localized receptive profiles of neurons in the early visual areas, and by the approximate translation invariance in their tuning. We inserted two main modifications to the standard model. First, we added a pre-filtering step that mimics the behavior of the LGN cells prior to the cortical processing. Second, we equipped the first convolutional layer with lateral connections defined by a diffusion law via a learned connectivity kernel, in analogy with the horizontal connectivity of V1. We focused on the CIFAR-10 dataset (Krizhevsky et al., 2009), since it contains natural images with a large statistics of orientations and shapes (see e.g., Ernst et al., 2007). We expected to find a strict similarity between the connectivity associated with this kernel and the one observed by Sanguinetti et al. (2010), since they are both learned from the statistics of natural images.

### 3.1. LGN in a CNN

As described in Bertoni et al. (2022), since the LGN pre-processes the visual stimulus before it reaches V1, we aim to introduce a “layer 0” that mimics this behavior. To this end, a convolutional layer ℓ^0^ composed by a single filter Ψ^0^ of size *s*^0^ × *s*^0^, a ReLU function and a batch normalization step is added at the beginning of the CNN architecture. If ℓ^0^ behaves similarly to the LGN, then Ψ^0^ should eventually attain a rotational invariant pattern, approximating the classical Laplacian-of-Gaussian (LoG) model for the RPs of LGN cells. In such a scenario, the rotational symmetry should emerge spontaneously during the learning process induced by the statistics of natural images, in analogy with the plasticity of the brain. In section 5 we will display the filter Ψ^0^ obtained after the training of the network and test its invariance under rotations.

### 3.2. Horizontal Connectivity of V1 in a CNN

Although the analogy with biological vision is strong, the feedforward mechanism implemented in CNNs is a simplified one, overlooking many of the processes contributing toward the interpretation of a visual scene. Our aim is to insert a simple mechanism of horizontal propagation defined by entirely learned connectivity kernels, and to analyze the invariance patterns, if any, arising as a result of learning from natural images.

Horizontal connections of convolutional type have been introduced in previous work through a recurrent formula, describing an evolution in time (Liang and Hu, 2015; Spoerer et al., 2017). However, the lateral kernels employed in these works are very localized, so that the connectivity kernel applied at each step only captures the connections between neurons very close-by in space. Obtaining a reconstruction of the long-range connectivity matrix resulting from the iteration of this procedure is not straightforward, since each step is followed by a nonlinear activation function. Here, we propose a formula defined as a simplified version of Equation (4), where the neural activity is updated through convolution with a connectivity kernel *K*^1^ with a wider support.The output ${\tilde{h}}^{1}$ is defined by averaging between the purely feedforward activation *h*^1^ = *ReLU*(*z*^1^) and the propagated activation *K*^1^ * *h*^1^, so that our update rule reads:


(7) $$\begin{array}{l} {\tilde{h}}^{1}=\frac{1}{2}h^{1}\;+\;K^{1}*h^{1}. \end{array}$$

Up to constants, this can be written as a discretization of a particular case of Equation (4), where the activation function *s* is linear.

The connectivity kernel *K*^1^ is a 4-dimensional tensor parameterized by 2-D spatial coordinates (*i, j*) and by the indices (*f, g*) corresponding to all pairs of ℓ^1^ filters. For fixed *f* and *g*, the function


(8) $$\begin{array}{l} \left(i,j\right)\mapsto K^{1}\left(i,j,f,g\right) \end{array}$$

represents the strength of connectivity between the filters Ψ_*f*_ and Ψ_*g*_, where the spatial coordinates indicate the displacement in space between the two filters. The intuitive idea is that *K*^1^ behaves like a “transition kernel” on the feature space of the first layer, modifying the feedforward output according to the learned connectivity between filters: the activation of a filter encourages the activation of other filters strongly connected with it.

### 3.3. Description of the Architecture and Training Parameters

In this section we give a detailed overview of our CNN architecture, as well as the data and training scheme employed.

**Figure 2:**
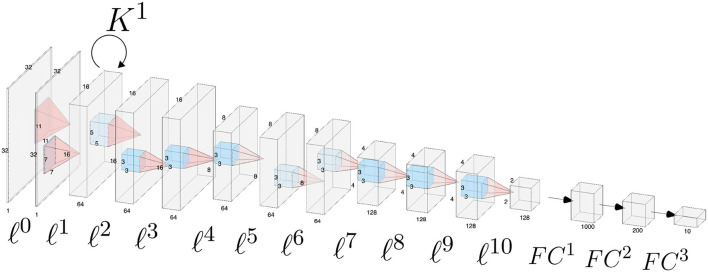
Schematic of the CNN architecture used for the analyses throughout the paper. Convolutional layers are numbered from ℓ^0^ to ℓ^10^, fully connected classification layers are denoted as *FC*^1^, *FC*^2^, *FC*^3^. Horizontal connections governed by the connectivity kernel *K*^1^ are represented as an operator acting on the feature space associated with layer ℓ^1^.

The architecture is composed by 11 convolutional layers, each one followed by a ReLU function and a batch normalization layer, and by three fully-connected layers. Appropriate zero padding was applied in order to keep the spatial dimensions unchanged after each convolutional layer.The layers composing the network architecture are displayed in Figure 2 and listed below. Convolutional layers are denoted by ℓ^1^, …, ℓ^10^, whereas fully connected layers are denoted by *FC*^1^, *FC*^2^, *FC*^3^. For simplicity we omit the ReLU function and the batch normalization layer.

**Table:** 

ℓ^0^	LGN layer: a single filter Ψ^0^ of size 11 × 11;
ℓ^1^	64 filters of size 7 × 7;
	lateral connectivity kernel *K*^1^ of size 13 × 13 × 64 × 64;
	max pooling of square size 2;
ℓ^2^	64 filters of size 5 × 5 × 64;
ℓ^3^, ℓ^4^	64 filters of size 3 × 3 × 64;
	max pooling of square size 2;
ℓ^5^, ℓ^6^	64 filters of size 3 × 3 × 64;
ℓ^7^	128 filters of size 3 × 3 × 64;
	max pooling of square size 2;
ℓ^8^, ℓ^9^, ℓ^10^	128 filters of size 3 × 3 × 128;
	max pooling of square size 2;
*FC*^1^, *FC*^2^, *FC*^3^	1,000, 200, and 10 output units respectively.

Since we wanted a gray scale image to be the input of our neural network, we transformed the CIFAR-10 dataset into gray scale images. All the parameters except the kernels *K*^1^ were first pre-trained in the absence of lateral connections for a maximum of 800 epochs, with early stopping when validation accuracy failed to increase for 80 consecutive epochs. We then inserted lateral connections in layer ℓ^1^, thus employing the full update rule in Equation (7), and implemented a further training stage: we re-updated the whole architecture including the lateral kernels *K*^1^ for a maximum of 800 epochs with early stopping as in the first phase. The initial purely feedforward training phase was intended both to obtain more stable learning of the receptive profiles, and to simulate the pre-existing orientation tuning of receptive profiles in V1 prior to the development of horizontal connections (Espinosa and Stryker, 2012). We stress however that, after this “initialization” stage, all the network weights were trained jointly: this allows for feedforward weights to possibly readjust in the presence of lateral connections.

In order to enhance the generalization ability of the network, along with the weight regularization and the batch normalization layers, we applied dropout with dropping probability equal to 0.5 after convolutional layer ℓ^10^. Dropout applied after the convolutional part of the network was intended to reduce overfitting in the final classification layers by weakening the reciprocal dependencies between weights of the same layer: this yields a more stable selection of the features relevant for image classification (Srivastava et al., 2014). We also applied dropout with dropping probability 0.2 when applying the kernel *K*^1^ (see also Semeniuta et al., 2016). Randomly dropping 20% lateral connections at each weight update had the purpose of avoiding co-adaptation of the connectivity weights, thus reinforcing their dependency on the intrinsic geometric properties of the receptive profiles. Applying dropout increased the performance on the test set from 72.24 to 80.28%. The network architecture and optimization procedure were coded using Pytorch (Paszke et al., 2017). The performance of the network was quantified as accuracy (fraction of correctly predicted examples) on the CIFAR-10 testing dataset.

**Figure 3:**
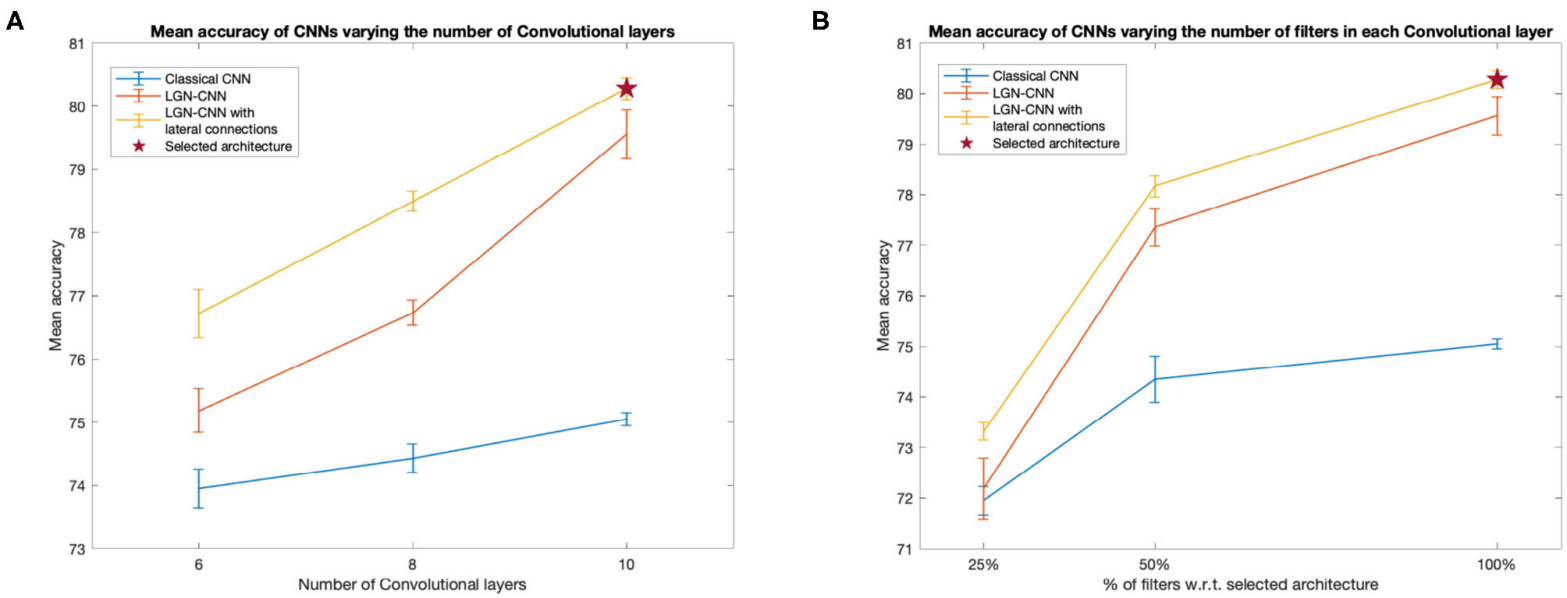
Behavior of the mean testing accuracy w.r.t. the number of layers **(A)** and units **(B)**. We compare the classical CNNs (without ℓ^0^ and *K*^1^ - blue line) with the LGN-CNN (with ℓ^0^ but without *K*^1^ - red line and with the LGN-CNN with lateral connections (as well as ℓ^0^ - yellow line). Error bars represent the standard error of the mean over 20 trainings. The selected model used throughout the paper is the one marked by a red star in both plots. **(A)** The *x*-axis represents the number of “standard” convolutional layers. **(B)** The *x*-axis represents the number of filters in each convolutional layer, expressed as a percentage w.r.t. the number of filters in the selected model.

We compared the performances of a classical CNN, an LGN-CNN with ℓ^0^ filtering and an LGN-CNN with ℓ^0^ filtering and the kernel *K*^1^. The comparison is aimed to understand the role of each of these layers in the accuracy of image recognition. We trained several CNNs for each of the previous configurations, varying the number of layers and units of the main CNN structure. Figure 3 summarizes the observed behavior of the mean testing accuracy. Figure 3A plots the accuracy against the number of standard convolutional layers (i.e., not counting ℓ^0^ or lateral connections). Figure 3B compares the mean performance of the model detailed above with architectures including only 25 and 50% the number of convolutional filters in each layer (e.g., each curve shows accuracy values for models that have 16, 32, and 64 filters in ℓ^1^, respectively). It is interesting to note that in each of these tests the performances of LGN-CNN with lateral connectivity and ℓ^0^ layer were better than the performances of LGN-CNN with only ℓ^0^ layer, and better than the classical CNN (without ℓ^0^ layer, or kernel *K*^1^). In particular, the role of the ℓ^0^ filter seems to be particularly interesting while testing accuracy w.r.t. the number of units (Figure 3B). Indeed, in this case the behavior of LGN-CNN with or without lateral connectivity kernel seems comparable, but both outperform the classical CNN. On the other hand the presence of the *K*^1^ kernel seems to be important to increase performances while testing accuracy w.r.t. the number of layers (Figure 3A). As expected, increasing the number of convolutional layers led to better performance for all three configurations (see Figure 3A). The same happens when varying the number of units of each convolutional layer (see Figure 3B). For the analyses described in the following, we selected the architecture that reached the best mean performances (80.28%± 0.17 over 20 trained instances of the model), i.e., the one detailed above and marked by a red asterisk in both plots.

We want to outline that, since we were interested in the emergence of symmetries, we did not focus on reaching state-of-the-art performances on classifying the CIFAR-10 dataset. However, our architecture reaches fair performances that can be increased by adding further convolutional layers and/or increasing the number of units. We prefer to keep a “simple enough” network structure allowing for short training times, even though the increase in performance did not reach a *plateau* in terms of number of layers and units.We stress that all the architectures examined showed a comparable behavior as regards the invariance properties of the convolutional layers ℓ^0^ and ℓ^1^ and the connectivity kernel *K*^1^. Therefore, it would be reasonable to expect a similar behavior also when further increasing the complexity of the model.

## 4. Emergence of Rotational Symmetry in the LGN Layer

As described in Bertoni et al. (2022) the introduction of a convolutional layer ℓ^0^ containing a single filter Ψ^0^ mimics the role of the LGN that pre-filters the input visual stimulus before it reaches the V1 cells. The authors have shown that a rotational invariant pattern is attained by Ψ^0^ for a specific architecture, suggesting that it should happen also for deeper and more complex architectures.

**Figure 4:**
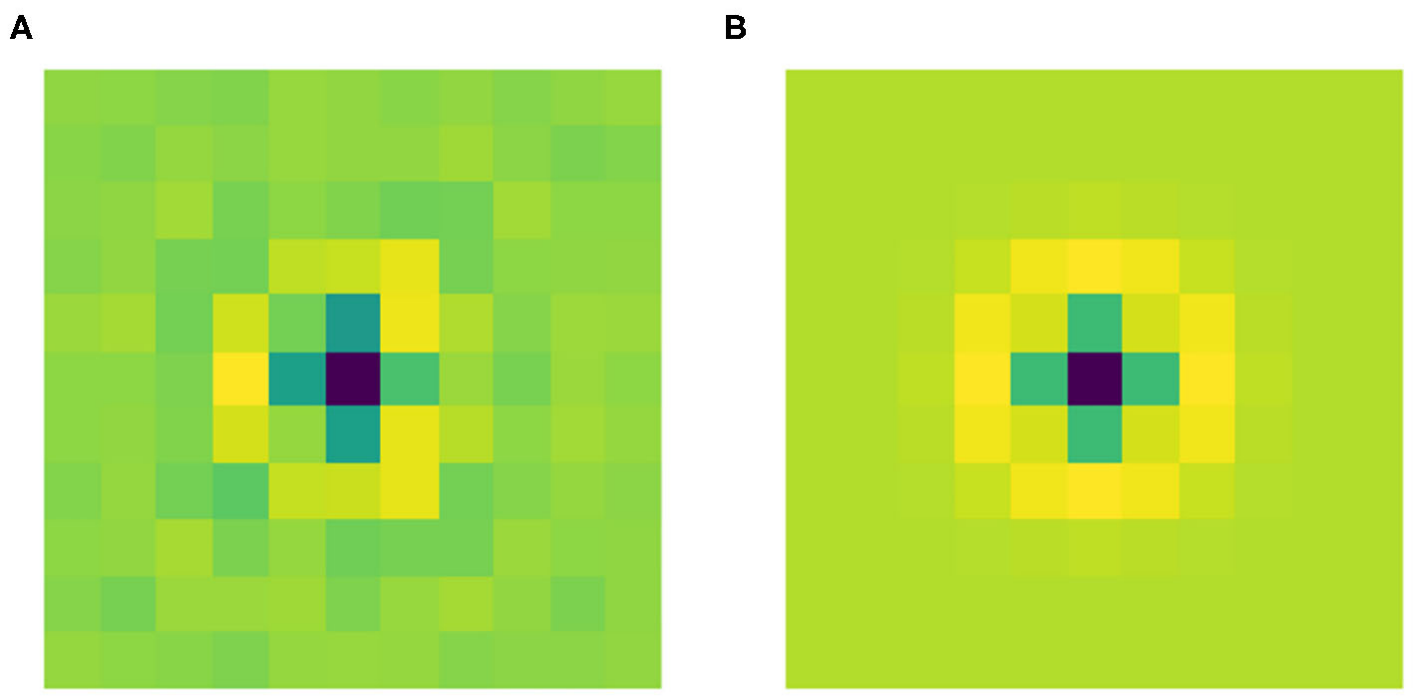
**(A)** The learned filter Ψ^0^ of the current architecture. **(B)** Its approximation as a LoG, with optimal σ = 0.184, yielding a correlation of 93.67% with the learned filter.

Figure 4A shows the filter Ψ^0^ obtained after the training phase. As expected it has a radially symmetric pattern and its maximum absolute value is attained in the center. Thus, it can be approximated by the classical LoG model for the RP of an LGN cell by finding the optimal value for the parameter σ in Equation (2). We used the built-in function *optimize.curve_fit* from the Python library SciPy. Figure 4B shows this approximation with σ = 0.184. Applying the built-in function *corrcoef* from the Python library NumPy, it turns out that the two functions have a high correlation of 93.67%.

These results show that Ψ^0^ spontaneously evolves into a radially symmetric pattern during the training phase, and more specifically its shape approximates the typical geometry of the RPs of LGN cells.

## 5. Emergence of Gabor-Like Filters in the First Layer

As introduced in section 2.2, the RPs of V1 simple cells can be modeled as Gabor functions by Equation (3). Moreover, the first convolutional layer of a CNN architecture usually shows Gabor-like filters (see e.g., Serre et al., 2007), assuming a role analogous to V1 orientation-selective cells. In this section, we first approximate the filters of ℓ^1^ as a bank of Gabor filters, in order to obtain a parameterization in terms of position (*x*_0_, *y*_0_) and orientation θ. This will provide a suitable set of coordinates for studying the corresponding lateral kernel in the ℝ^2^ × *S*^1^ domain, see section 6.

### 5.1. Approximation of the Filters as Gabor Functions

**Figure 5:**
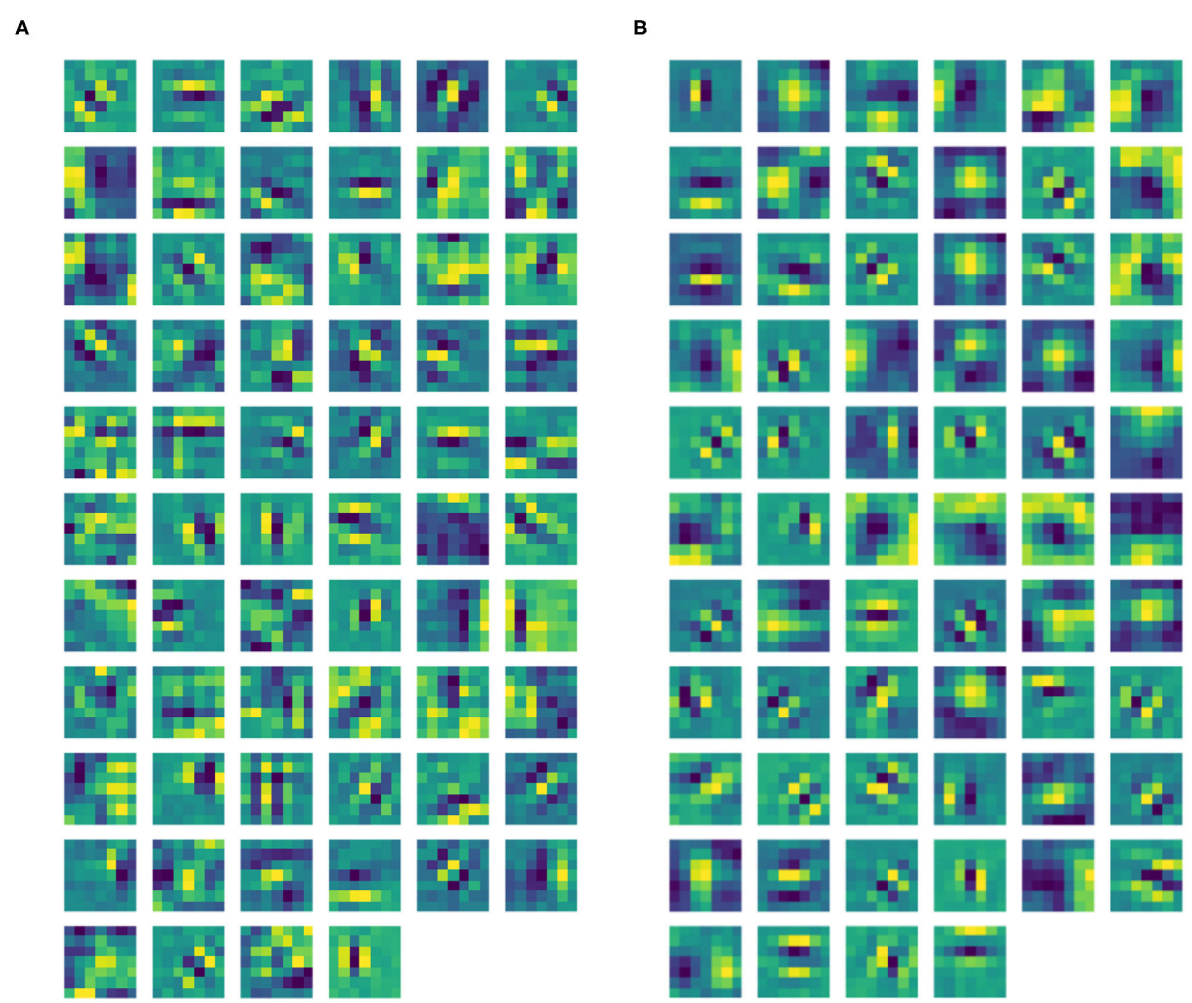
**(A)** Learned filters of ℓ^1^ of our CNN architecture. **(B)** Learned filters of ℓ^1^ of the same CNN architecture, but without ℓ^0^.

After the training phase, Gabor-like filters emerge in ℓ^1^ as expected (see Figure 5A). The filters in ℓ^1^ were approximated by the Gabor function in Equation (3), where all the parameters were found using the built-in function *optimize.curve_fit* from the Python library SciPy. The mean Pearson's correlation coefficient (obtained using the built-in function *pearsonr* from the Python library SciPy) between the filters and their Gabor approximations was 89.14%. All correlation values were statistically significant (*p* < .001). Thanks to the introduction of the pre-filtering ℓ^0^, our layer ℓ^1^ only contains filters sharply tuned for orientation, with no Gaussian-like filters. Indeed, by removing ℓ^0^, the two types of filters are mixed up in the same layer. See Figure 5B, showing the ℓ^1^ filters of a trained CNN with the same architecture, but without ℓ^0^. However, we can note from Figure 5A that some filters have more complex shapes that are neither Gaussian-, nor Gabor-like; indeed, since we have not introduced other geometric structures on following layers, it is reasonable to observe the emergence of more complex patterns.

**Figure 6:**
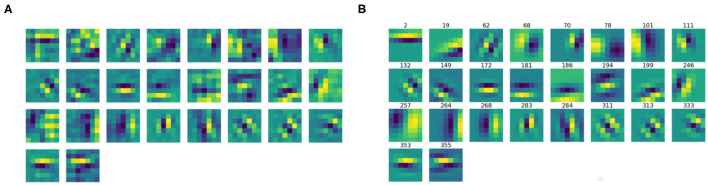
**(A)** The learned filters of ℓ^1^ with odd parity, ordered w.r.t. the orientation θ obtained from the Gabor approximation. **(B)** The approximating odd Gabor filters, labeled by their orientation.

**Figure 7:**
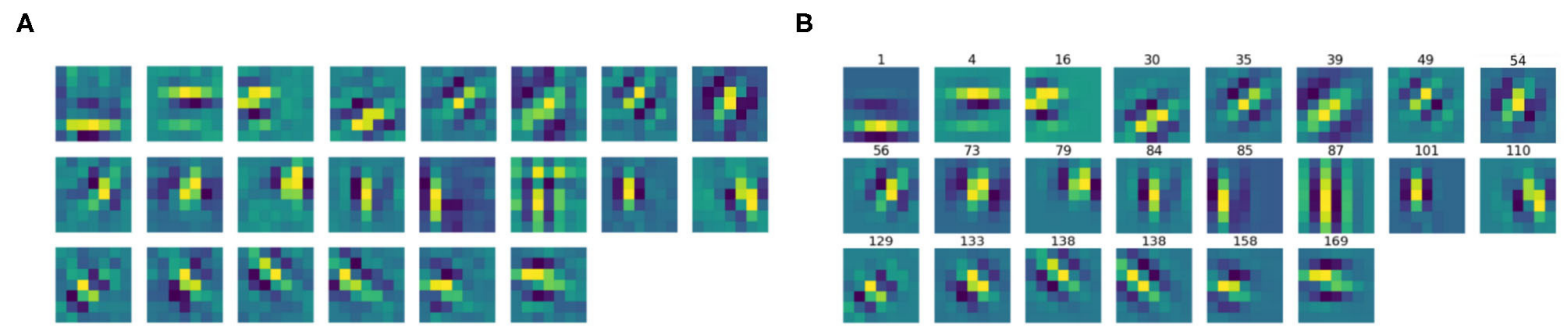
**(A)** The learned filters of ℓ^1^ with even parity, ordered w.r.t. the orientation θ obtained from the Gabor approximation. **(B)** The approximating even Gabor filters, labeled by their orientation.

We then split the filter bank w.r.t. the parity of their approximation, indicated by the parameter ϕ, that was forced to be between −π and π. Specifically, we labeled a filter as odd if $\frac{\pi }{4}<|\phi |<\frac{3\pi }{4}$, as even if $0<|\phi |<\frac{\pi }{4}$ or $\frac{3\pi }{4}<|\phi |<\pi$. Figures 6, 7 show the odd and even filters rearranged w.r.t. the orientation θ. For the sake of visualization, those even filters whose central lobe had negative values were multiplied by -1. The orientation values are quite evenly sampled, allowing the neural network to detect even small orientation differences. Most of the filters have high frequencies, allowing them to detect thin boundaries, but some low-frequency filters are still present. The interest of the organization in odd and even filters comes from comparison with neurophysiological data. Indeed, it is well known that the RPs of simple cells are organized in odd and even profiles, with different functionality. The odd ones are responsible for boundary detection, the even ones for the interior.

## 6. Emergence of Orientation-Specific Connectivity in the Horizontal Kernel

In this section, we will study the learned connectivity kernel *K*^1^, to investigate whether it shows any invariances compatible with the known properties of the lateral connectivity of V1. The connectivity kernel was re-parameterized as a function of relative position and orientation of the profiles. In the following, we first give details on this coordinate change. We then examine the short-range connectivity (see e.g., Bressloff and Cowan, 2003) defined by the learned interactions between learned profiles with different preferred orientations belonging to the same hypercolumn (i.e., sharing the same retinal position) as a function of the relative orientation between the filters. We finally study the learned pattern of connectivity across both spatial positions and orientations, modeling the interaction between different hypercolumns.

### 6.1. Re-parameterization of the Connectivity Kernel Using the First Layer Approximation

In order to study the selectivity of the connectivity kernel to the tuning properties of ℓ^1^ filters, we first rearranged it based on the set of coordinates in ℝ^2^ × *S*^1^ induced by the Gabor approximation of the filters.

We first split the kernel w.r.t. the parity of ℓ^1^ filters, resulting in two separate connectivity kernels for even and odd filters. We then adjusted the spatial coordinates of each kernel using the estimated Gabor filter centers (*x*_0_, *y*_0_). Specifically, for each *f, g* in {1, …, *n*}, we shifted the kernel *K*^1^(·, ·, *f, g*) of Equation (8) so that a displacement of (*i, j*) = (0, 0) corresponds to the situation where the centers of the filters Ψ_*f*_ and Ψ_*g*_ coincide. Then, the original ordering of *f, g* in {1, …, *n*} has no geometric meaning. However, each filter Ψ_*f*_ is now associated with an orientation θ_*f*_ obtained from the Gabor approximation. Therefore, we rearranged the slices of *K*^1^ so that the *f* and *g* coordinates were ordered by the corresponding orientations θ_*f*_ and θ_*g*_. By fixing one filter Ψ_*f*_, we then obtained a 3-D kernel


(9) $$\begin{array}{l} \left(i,j,g\right)\mapsto K^{1}\left(i,j,f,g\right) \end{array}$$

defined on ℝ^2^ × *S*^1^, describing the connectivity between Ψ_*f*_ and all the other ℓ^1^ filters, each shifted by a set of local displacements (*i, j*) ∈ {−6, …, 6} × {−6, …, 6}.

### 6.2. Non-maximal Suppression Within Orientation Hypercolumns

The re-parameterized connectivity kernel *K*^1^ describes the strength of interaction as a function of relative displacement and relative orientation of the profiles. In this section, we restrict ourselves to the case of a displacement (*i, j*) = (0, 0), i.e., the case of profiles belonging to the same hypercolumn of orientation. We thus study, for fixed *f*, the function


(10) $$\begin{array}{l} \theta_{g}\mapsto K^{1}\left(0,0,f,g\right)=K^{1}\left(0,0,\theta_{f},\theta_{g}\right). \end{array}$$

**Figure 8:**
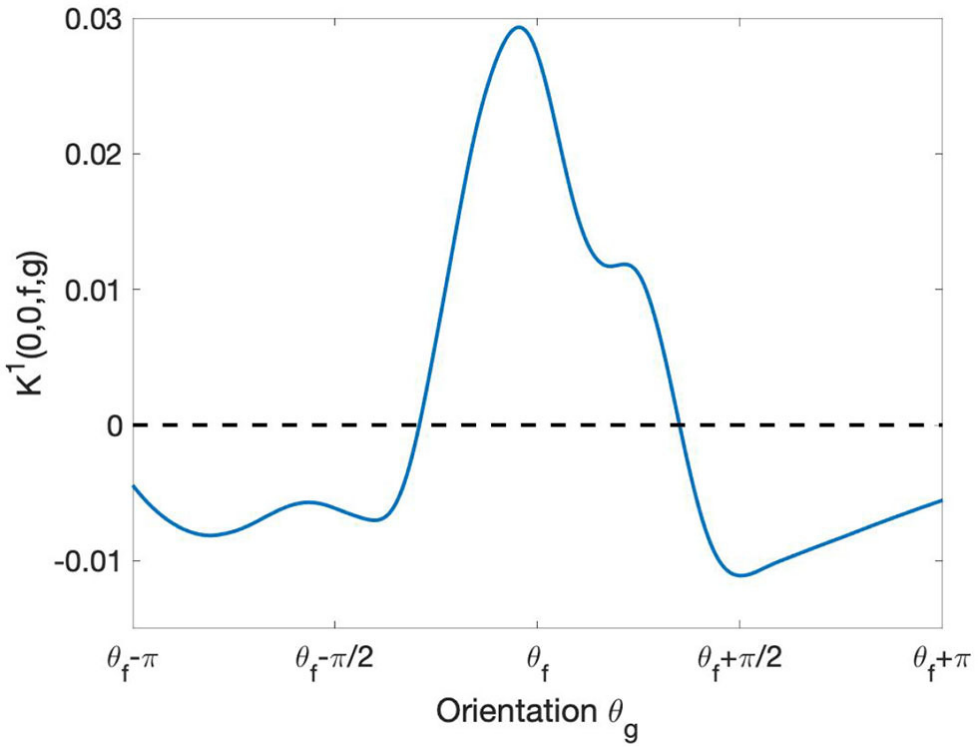
Behavior of learned short-range intracortical connections w.r.t. the relative orientation. The curve displays the strength of interaction between the filter Ψ_*f*_ with orientation $\theta_{f}=\frac{2\pi }{5}$ and the other filters Ψ_*g*_ centered at the same point [i.e., with relative displacement = (0, 0)] as a function of their orientation θ_*g*_. The curve has been smoothed using the MatLab built-in function *smoothdata*.

This function, plotted in Figure 8, describes the learned pattern of excitation and inhibition between the profiles Ψ_*f*_ and Ψ_*g*_ as a function of the orientation θ_*g*_. Notably, the resulting interaction profile showed a “Mexican hat”-like shape, indicating an enhancement of the response to the optimal orientation and a suppression of the response to non-optimal orientations. This type of profile has been shown to yield a sharpening of the orientation tuning (Bressloff and Cowan, 2003).

### 6.3. Association Fields Induced by the Connectivity Kernel

In this section, we will show the anisotropic distribution of learned connectivity weight w.r.t. spatial position and orientation. The interaction between profiles centered at different points of the retinal space models the connectivity linking distinct hypercolumns of orientation. This has been described geometrically in previous work as illustrated in section 2.2. In the following, we will compare the properties of our learned connectivity with some existing models.

Starting from the re-parameterized kernel centered around a filter Ψ_*f*_ as in Equation (9), we used the θ-coordinates to construct a 2-D association field as in Sanguinetti et al. (2010). We first defined a 2-D vector field by projecting down the orientation coordinates weighted by the kernel values. Specifically, for each spatial location (*i, j*), we defined


(11) $$\begin{array}{l} V\left(i,j\right):=\underset{g}{\max}K^{1}\left(i,j,f,g\right)\;\cdot \;\frac{\sum_{g=1}^{n}K^{1}\left(i,j,f,g\right)v_{g}}{\left||\sum_{g=1}^{n}K^{1}\left(i,j,f,g\right)v_{g}|\right|}, \end{array}$$

where $v_{g}\in {\mathbb{R}}^{2}$ is a unitary vector with orientation θ_*g*_. This yields for each point (*i, j*) a vector whose orientation is essentially determined by the leading θ values in the fiber, i.e., the ones were the kernel has the highest values. The norm of the vector is determined by the maximum kernel value over (*i, j*). Finally, we defined the association field as the integral curves of the so-obtained vector field *V* starting from points along the trans-axial direction in a neighborhood of (0, 0).

**Figure 9:**
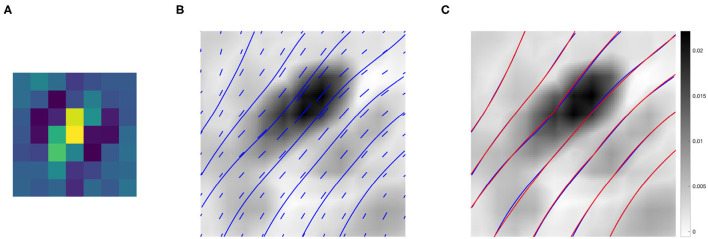
**(A)** The 7 × 7 even filter Ψ_*f*_, with orientation $\theta_{f}=138^{∙}$. **(B)** Projection on the (*x, y*) plane of the connectivity kernel computed around the filter Ψ_*f*_ displayed in **(A)**. The projection is obtained by maximizing the 3-D kernel of Equation (9) over the variable *g*, and the values are color-coded from white (low) to black (high). For better visualization, the kernel has been upsampled by a factor of 10 using the built-in Matlab function *imresize*. The vector field *V* and its integral curves forming the induced association field are shown in blue over the intensity values. **(C)** The association field induced by the kernel of even filters around Ψ_*f*_ (blue), and its approximation using the integral curves defined in Equation (6) (red)—displayed over the kernel projection.

Figure 9B shows the association field obtained from the kernel computed around the filter Ψ_*f*_ in Figure 9A, with orientation $\theta_{f}=\frac{3\pi }{10}$. The vector field *V* was plotted using the Matlab function *quiver*, and the integral curves were computed using the Matlab function *streamline*. The vectors and curves are plotted over a 2-D projection of the kernel obtained as follows. The kernel was first resized by a factor of 10 using the built-in Matlab function *imresize* for better visualization, and then projected down on the spatial coordinates by taking the maximum over *g*. Note that around (0, 0) the field is aligned with the orientation of the starting filter Ψ_*f*_, while it starts to rotate when it moves away from the center – consistently with the typical shape of psychophysical association fields, see section 2.2.

We also outline the similarity with the integral curves of Sanguinetti et al. (2010), see Figure 1D. However, in our case the rotation is less evident since the spatial displacement encoded in the kernel *K*^1^ is more localized than the edge co-occurrence kernel constructed in Sanguinetti et al. (2010).

Figure 9C shows in red the approximation of each integral curve as a circular arc, obtained by fitting the parameter *k* of Equation (6) to minimize the distance between the two curves. The empirical curves induced by the learned connectivity kernel were very close to the theoretical curves, with a mean Euclidean distance of 0.0036.

## 7. Discussion

In this work, we showed how approximate group invariances arise in the early layers of a biologically inspired CNN architecture during learning on natural images, and we established a parallel with the architecture and plasticity of the early visual pathway.

In standard CNN architectures, the LGN processing stage is incorporated into the modeled early cortical processing, thus making it impossible to disjointedly analyzes the different symmetries arising in these two stages. In our work, we were interested in *decomposing* the first network layer transformation in order to separately model the LGN analysis, which is a crucial step in the processing of a visual input. Indeed, it is well established (see e.g., Levine and Cleland, 2001; Uglesich et al., 2009) that the average firing rate of the retinal ganglion cells is much higher than that of LGN cells. This difference of firing rate could probably be related to the structure of synaptic connection between RGB and LGN, and its study would require a further decomposition of the architecture of the CNN, which could be object of studies in a future work. As pointed out in Rathbun et al. (2010), retinogeniculate processing increases sparseness in the neural code by selecting the most informative spikes to the visual cortex, thus providing a resampled map of visual space (Martinez et al., 2014). The subsequent partial loss of information can be partly reconstructed via lateral connections. From a biological point of view, this information compression is necessary due to the limited size of the nerves that carry the impulse from the LGN to V1. Therefore, the role of the LGN in perception reaches beyond that of a mere relay area, and it is worth individual attention. Notably, the pre-filtering stage ℓ^0^ inserted in our architecture developed during the network training to give rise to a LoG-shaped filter, closely resembling the typical radially symmetric LGN receptive profile (see also Bertoni et al., 2022). In addition, the presence of ℓ^0^ enhanced the orientation tuning of the filters in the first convolutional layer ℓ^1^. The emergence of orientation-selective first-layer profiles was consistent with the results of previous studies analyzing feature selectivity in CNN layers; the separation of the image elaboration into two steps that may be roughly associated with sub-cortical and early cortical processing had the effect of sharpening their orientation tuning.

Receptive profiles in CNNs are modeled to be translation-invariant, essentially implementing an “ice-cube” representation of the hypercolumnar structure. Despite their limitations, CNNs provide a powerful abstraction, since even in such a simplified setting we observe the emergence of biologically relevant symmetries. As a future direction, it would be interesting to expand the current study to include the learning of feature maps. This may be done by restricting the layer response to a (learned) lower-dimensional space, thus allowing to observe data-driven feature maps and investigate on other phenomena such as the development of a radial bias (see e.g., Philips and Chakravarthy, 2017).

Further, the introduction of plastic lateral kernels in the first network layer allowed us to investigate how the (initially random) connectivity evolved during the training to optimize image recognition. Our lateral connections take the form of a linear diffusion step governed by a convolutional kernel *K*^1^. We were mainly interested in studying the relation between the learned weights *K*^1^, expressing the connectivity between filters, and the relative tuning of the corresponding filters. The Gabor approximation of first layer receptive profiles provided us with a set of coordinates to re-map the kernel *K*^1^ into the ℝ^2^ × *S*^1^ feature space, thus allowing to express the connectivity strength directly in terms of relative positions and orientations of the filters. The learned short-range connections between cells sharing the same spatial position but different orientation revealed a “Mexican hat”-like interaction profile. This arrangement of excitation and inhibition suppressing the response to non-optimal orientations has been shown to yield a sharpening of the orientation tuning (Bressloff and Cowan, 2003). We then analyzed the learned pattern of connectivity across both spatial positions and orientations, modeling the interaction between different hypercolumns.From the distribution of kernel values over orientations, we constructed association fields induced by the learned connectivity as a vector field on ℝ^2^. The integral curves of the vector field turned out to link preferentially those pairs of neurons whose relative position and orientation tuning formed a collinear or co-circular pattern.Strikingly, our association fields bear a close resemblance to those obtained from edge co-occurrence in natural images by Sanguinetti et al. (2010). We stress that such geometric properties arose spontaneously during the training of the CNN architecture on a dataset of natural images, the only constraint being the translation-invariance imposed by the convolutional structure of the network.

The learned connectivity kernel *K*^1^ describes the strength of interactions between receptive profiles shifted by up to 6 pixels w.r.t. one another in each direction. A wider connectivity may be modeled by taking further propagation steps. Thanks to the linearity, this composition would be equivalent to taking one step of propagation with the long-range kernel 2*K*^1^ + *K*^1^ * *K*^1^, obtained via “self-replication” from *K*^1^ through convolution against itself. Indeed, iteration of the update rule yields:


$$\begin{array}{l} \frac{1}{2}\left({\tilde{h}}^{1}\;+\;K^{1}*{\tilde{h}}^{1}\right)=\frac{1}{4}\left(h^{1}\;+\;2K^{1}*h^{1}\;+\;K^{1}*K^{1}*h^{1}\right). \end{array}$$

Therefore, a promising direction for future work could be to consider larger natural images and examine wider horizontal connectivity obtained via multiple diffusion steps—as well as to compare the propagated long-range connectivity kernels with theoretical kernels.

As other future perspectives, we would like to extend our study by considering a wider range of features—possibly leading to a finer characterization of the geometry of first layer filters, including more complex types of receptive profiles. Finally, another direction could be to extend our model by comparing the properties of deeper network layers to the processing in higher cortices of the visual system.

## Data Availability Statement

Publicly available datasets were analyzed in this study. This data can be found at: CIFAR-10 dataset Krizhevsky et al. (2009).

## Author Contributions

All authors listed have made a substantial, direct and intellectual contribution to the work, and approved it for publication.

## Funding

This work was supported by progetto alte competenze per la ricerca e il trasferimento tecnologico POR FSE 2014/2020 Obiettivo tematico 10. FB: this project has received funding from the European Union's Horizon 2020 research and innovation program under the Marie Skłodowska-Curie Grant Agreement No 754362. AS and GC: this project has received funding from GHAIA - Marie Skłodowska-Curie Grant Agreement No. 777822.


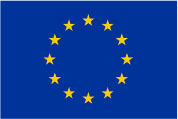


## Conflict of Interest

The authors declare that the research was conducted in the absence of any commercial or financial relationships that could be construed as a potential conflict of interest.

## Publisher's Note

All claims expressed in this article are solely those of the authors and do not necessarily represent those of their affiliated organizations, or those of the publisher, the editors and the reviewers. Any product that may be evaluated in this article, or claim that may be made by its manufacturer, is not guaranteed or endorsed by the publisher.

## References

[B1] Abbasi-Sureshjani S. Favali M. Citti G. Sarti A. ter Haar Romeny B. M. (2018). Curvature integration in a 5d kernel for extracting vessel connections in retinal images. IEEE Trans. Image Process. 27, 606–621. 10.1109/TIP.2017.2761543 28991743

[B2] Anselmi F. Leibo J. Z. Rosasco L. Mutch J. Tacchetti A. Poggio T. (2016). Unsupervised learning of invariant representations. Theor. Comp. Sci. 633, 112–121. 10.1016/j.tcs.2015.06.048

[B3] Barbieri D. Cocci G. Citti G. Sarti A. (2014). A cortical-inspired geometry for contour perception and motion integration. J. Math. Imaging Vis. 49, 511–529. 10.1007/s10851-013-0482-z

[B4] Bertoni F. Citti G. Sarti A. (2022). LGN-CNN: a biologically inspired CNN architecture. Neural Networks. 145, 42–55. 10.1016/j.neunet.2021.09.024 34715534

[B5] Bosking W. Zhang Y. Schoenfield B. Fitzpatrick D. (1997). Orientation selectivity and the arrangement of horizontal connections in tree shrew striate cortex. J. Neurosci. 17, 2112–2127. 10.1523/JNEUROSCI.17-06-02112.1997 9045738 PMC6793759

[B6] Bressloff P. C. Cowan J. D. (2003). The functional geometry of local and long-range connections in a model of V1. J. Physiol. Paris 97, 221–236. 10.1016/j.jphysparis.2003.09.017 14766143

[B7] Citti G. Sarti A. (2006). A cortical based model of perceptual completion in the roto-translation space. J. Math. Imaging Vis. Arch. 24, 307–326. 10.1007/s10851-005-3630-2

[B8] Daugman J. G. (1985). Uncertainty relation for resolution in space, spatial frequency, and orientation optimized by two-dimensional visual cortical filters. J. Opt. Soc. Am. 2, 1160–1169. 10.1364/JOSAA.2.001160 4020513

[B9] Ernst U. Deneve S. Meinhardt G. (2007). Detection of gabor patch arrangements is explained by natural image statistics. BMC Neurosci. 8:P154. 10.1186/1471-2202-8-S2-P154

[B10] Espinosa J. S. Stryker M. P. (2012). Development and plasticity of the primary visual cortex. Neuron 75, 230–249. 10.1016/j.neuron.2012.06.009 22841309 PMC3612584

[B11] Field D. J. Hayes A. Hess R. F. (1993). Contour integration by the human visual system: evidence for a local association field. Vision Res. 33, 173–193. 10.1016/0042-6989(93)90156-Q 8447091

[B12] Girosi F. Jones M. Poggio T. (1995). Regularization theory and neural networks architectures. Neural Comput. 7, 219–269. 10.1162/neco.1995.7.2.219

[B13] Hoffman W. (1989). The visual cortex is a contact bundle. Appl. Math. Comput. 32, 137–167. 10.1016/0096-3003(89)90091-X110851

[B14] Hubel D. H. (1987). Eye, Brain, and Vision. New York, NY: WH Freeman (Scientific American Library).

[B15] Hubel D. H. Wiesel T. N. (1962). Receptive fields, binocular interaction and functional architecture in the cat visual cortex. J. Physiol. 160, 106–154. 10.1113/jphysiol.1962.sp006837 14449617 PMC1359523

[B16] Hubel D. H. Wiesel T. N. (1977). Ferrier lecture: functional architecture of macaque monkey visual cortex. Proc. R. Soc. Lond. B Biol. Sci. 198, 1–59. 10.1098/rspb.1977.0085 20635

[B17] Koenderink J. J. van Doom A. J. (1987). Representation of local geometry in the visual system. Biol. Cybern. 55, 367–375. 10.1007/BF00318371 3567240

[B18] Krizhevsky A. (2009). Learning multiple layers of features from tiny images. Technical report.

[B19] Lawrence S. Giles C. L. Tsoi A. C. Back A. D. (1997). Face recognition: a convolutional neural-network approach. IEEE Trans. Neural Netw. 8, 98–113. 10.1109/72.554195 18255614

[B20] LeCun Y. Bottou L. Bengio Y. Haffner P. (1998). Gradient-based learning applied to document recognition. Proc. IEEE 86, 2278–2324. 10.1109/5.72679127295638

[B21] Lee T. S. (1996). Image representation using 2d Gabor wavelets. IEEE Trans. Pattern Anal. Mach. Intell. 18, 959–971. 10.1109/34.54140627295638

[B22] Levine M. W. Cleland B. G. (2001). An analysis of the effect of retinal ganglion cell impulses upon the firing probability of neurons in the dorsal lateral geniculate nucleus of the cat. Brain Res. 902, 244–254. 10.1016/s0006-8993(01)02411-8 11384618

[B23] Liang M. Hu X. (2015). Recurrent convolutional neural network for object recognition, in 2015 IEEE Conference on Computer Vision and Pattern Recognition (CVPR) (Boston, MA: IEEE).

[B24] Martinez L. M. Molano-Mazón M. Wang X. Sommer F. T. Hirsch J. A. (2014). Statistical wiring of thalamic receptive fields optimizes spatial sampling of the retinal image. Neuron 81, 943–956. 10.1016/j.neuron.2013.12.014 24559681 PMC4114508

[B25] Montobbio N. Bonnasse-Gahot L. Citti G. Sarti A. (2019). Kercnns: biologically inspired lateral connections for classification of corrupted images. CoRR, abs/1910.08336.

[B26] Montobbio N. Sarti A. Citti G. (2020). A metric model for the functional architecture of the visual cortex. J. Appl. Math. 80, 1057–1081. 10.1137/18M120141X

[B27] Paszke A. Gross S. Chintala S. Chanan G. Yang E. DeVito Z.. (2017). Automatic differentiation in pytorch, in NIPS-W. California, CA

[B28] Petitot J. (2008). Neurogéométrie de la vision - *Modèles mathématiques et physiques des architectures fonctionnelles*. Paris: Éditions de l'École Polytechnique.

[B29] Petitot J. Tondut Y. (1999). Vers une neuro-géométrie. Fibrations corticales, structures de contact et contours subjectifs modaux. Math. Inform. Sci. Hum. 145, 5–101. 10.4000/msh.2809

[B30] Philips R. T. Chakravarthy V. S. (2017). A global orientation map in the primary visual cortex (V1): could a self organizing model reveal its hidden bias? Front. Neural Circ. 10:109. 10.3389/fncir.2016.00109 28111542 PMC5216665

[B31] Poggio T. Anselmi F. (2016). Visual Cortex and Deep Networks: Learning Invariant Representations. Cambridge, MA: The MIT Press.

[B32] Rathbun D. L. Warland D. K. Usrey W. M. (2010). Spike timing and information transmission at retinogeniculate synapses. J. Neurosci. 30, 13558–13566. 10.1523/JNEUROSCI.0909-10.2010 20943897 PMC2970570

[B33] Sanguinetti G. Citti G. Sarti A. (2008). Image completion using a diffusion driven mean curvature flowin a sub-riemannian space, in Proceedings of the Third International Conference on Computer Vision Theory and Applications (Madeira), 46–53.

[B34] Sanguinetti G. Citti G. Sarti A. (2010). A model of natural image edge co-occurrence in the rototranslation group. J. Vis. 10:37. 10.1167/10.14.37 21196513

[B35] Sarti A. Citti G. (2015). The constitution of visual perceptual units in the functional architecture of V1. J. Comput. Neurosci. 38, 285–300. 10.1007/s10827-014-0540-6 25529294

[B36] Sarti A. Citti G. Petitot J. (2008). The symplectic structure of the primary visual cortex. Biol. Cybern. 98, 33–48. 10.1007/s00422-007-0194-9 18008082

[B37] Semeniuta S. Severyn A. Barth E. (2016). Recurrent dropout without memory loss, in Proceedings of COLING 2016, the 26th International Conference on Computational Linguistics: Technical Papers (Osaka: The COLING 2016 Organizing Committee), 1757–1766.

[B38] Serre T. Wolf L. Bileschi S. Riesenhuber M. Poggio T. (2007). Robust object recognition with cortex-like mechanisms. IEEE Trans. Pattern Anal. Mach. Intell. 29, 411–426. 10.1109/TPAMI.2007.56 17224612

[B39] Sherstinsky A. (2020). Fundamentals of recurrent neural network (rnn) and long short-term memory (lstm) network. Physica D 404:132306. 10.1016/j.physd.2019.132306

[B40] Spoerer C. McClure P. Kriegeskorte N. (2017). Recurrent convolutional neural networks: a better model of biological object recognition. Front. Psychol. 8:1551. 10.3389/fpsyg.2017.01551 28955272 PMC5600938

[B41] Srivastava N. Hinton G. Krizhevsky A. Sutskever I. Salakhutdinov R. (2014). Dropout: a simple way to prevent neural networks from overfitting. J. Mach. Learn. Res. 15, 1929–1958. Available online at: https://jmlr.org/papers/v15/srivastava14a.html

[B42] Uglesich R. Casti A. Hayot F. Kaplan E. (2009). Stimulus size dependence of information transfer from retina to thalamus. Front. Syst. Neurosci. 3:10. 10.3389/neuro.06.010.2009 19838326 PMC2762372

[B43] Wilson H. R. Cowan J. D. (1972). Excitatory and inhibitory interactions in localized populations of model neurons. Biophys. J. 12, 1–24. 10.1016/S0006-3495(72)86068-5 4332108 PMC1484078

[B44] Yamins D. Hong H. Cadieu C. Dicarlo J. (2015). Hierarchical modular optimization of convolutional networks achieves representations similar to macaque it and human ventral stream, in Advances in Neural Information Processing Systems. Red Hook, NY.

[B45] Yamins D. L. K. DiCarlo J. J. (2016). Using goal-driven deep learning models to understand sensory cortex. Nat. Neurosci. 19, 356–365. 10.1038/nn.4244 26906502

[B46] Zeiler M. Fergus R. (2014). Visualizing and understanding convolutional networks, in Lecture Notes in Computer Science, Vol 8689,ECCV 2014, eds Fleet D. Pajdla B Schiele Tuytelaars T. (Cham: Springer), 1097–1105.

